# Do consumers' preferences for improved provision of malaria treatment services differ by their socio-economic status and geographic location? A study in southeast Nigeria

**DOI:** 10.1186/1471-2458-10-7

**Published:** 2010-01-05

**Authors:** Nkoli P Uguru, Obinna E Onwujekwe, Nnenna G Tasie, Benjamin S Uzochukwu, Uche E Ezeoke

**Affiliations:** 1 Health Policy Research Group, Department of Pharmacology and Therapeutics, College of Medicine, University of Nigeria, Enugu, Nigeria; 2 Department of Preventive Dentistry, Faculty of Dentistry, College of Medicine, University of Nigeria, Enugu, Nigeria; 3 Department of Health Administration and Management, College of Medicine, University of Nigeria, Enugu, Nigeria; 4 Department of Community Medicine, College of Medicine, University of Nigeria, Enugu, Nigeria; 5 London School of Hygiene and Tropical Medicine, London, UK

## Abstract

**Background:**

Improvement of utilization of malaria treatment services will depend on provision of treatment services that different population groups of consumers prefer and would want to use. Treatment of malaria in Nigeria is still problematic and this contributes to worsening burden of the disease in the country. Therefore this study explores the socio-economic and geographic differences in consumers' preferences for improved treatment of malaria in Southeast Nigeria and how the results can be used to improve the deployment of malaria treatment services.

**Methods:**

This study was undertaken in Anambra state, Southeast Nigeria in three rural and three urban areas. A total of 2,250 randomly selected householders were interviewed using a pre tested interviewer administered questionnaire. Preferences were elicited using both a rating scale and ranking of different treatment provision sources by the respondents. A socio-economic status (SES) index was used to examine for SES differences, whilst urban-rural comparison was used to examine for geographic differences, in preferences.

**Results:**

The most preferred source of provision of malaria treatment services was public hospitals (30.5%), training of mothers (19%) and treatment in Primary healthcare centres (18.1%). Traditional healers (4.8%) and patent medicine dealers (4.2%) were the least preferred strategies for improving malaria treatment. Some of the preferences differed by SES and by a lesser extent, the geographic location of the respondents.

**Conclusion:**

Preferences for provision of improved malaria treatment services were influenced by SES and by geographic location. There should be re-invigoration of public facilities for appropriate diagnosis and treatment of malaria, in addition to improving the financial and geographic accessibility of such facilities. Training of mothers should be encouraged but home management will not work if the quality of services of patent medicine dealers and pharmacy shops where drugs for home management are purchased are not improved. Therefore, there is the need for a holistic improvement of malaria treatment services.

## Background

Malaria is a major cause of mortality and morbidity in Nigeria, and is responsible for 30% of childhood mortality, 11% of maternal mortality and more than 60% of out-patient visits [1]. This disease has also impacted negatively on the Nation's economy so much so that Nigeria loses about 132 billion Naira annually to the disease [2]. One of the key strategies endorsed by the Abuja meeting was to take actions to ensure that by 2005 at least 60% of those suffering from malaria have prompt access to appropriate and affordable treatment and are able to initiate treatment within 24 hours of the onset of symptoms. This was termed effective management of malaria nearer the home and was thus adopted as another strategy to combat malaria [3].

The limited healthcare facilities that exist in rural Nigeria make it difficult to provide the required good quality malaria management services [4,5]. Poor quality of treatment amongst other factors has shown to be contributory to the ineffective control of malaria [6-10]. Similarly, some other studies have shown that the public and private sector are usually ineffective in managing malaria because of inappropriate prescription, lack of drugs and diagnostic facilities [6,11]

The preferences of consumers for sources for improving treatment of malaria should be understood and used to improve provision and utilization of appropriate malaria treatment services. Peoples' perception of the ease of accessing the various providers of malaria treatment can potentially determine their health-seeking behavior [12] and by extension their preferences for different providers. Majority of households named the hospital as their preferred choice for malaria treatment, with the availability of drugs and trained personnel as very important reasons for their preferences [13].

There is need to increase the body of knowledge about the link of socio-economic status to consumers' preferences for improved malaria treatment, for proving appropriate and timely treatment of malaria. However, some strategies have been suggested which will be useful for providing timely, appropriate, and potentially equitable management of malaria within communities these include health education to mothers [5,14,15]; and the use of village or community health workers [16,17].

Many of the recommended interventions for improving appropriate treatment of malaria could be at variance with what the communities really prefer. This will lead to poor utilization of such services thereby negating the original ideas behind their deployment. The ability for successful and sustainable disease control programs depends very much on "listening to the people" [18]. Therefore it is better to determine consumers' preferences and needs and use the information to guide program design because imposed programs invariably fail as they reflect professional views and may not meet the priorities and aspirations of communities [19].

This study examined the preferences for improved malaria treatment by consumers and disaggregates these preferences by socio-economic status and geographic location of the respondents. The study also compared whether the rating and ranking scales will produce similar results, so as to assure the internal validity of the preferences. The findings are useful for evidence-based policy-making and development of strategies for equitably improving the deployment of demand-responsive malaria treatment services in different geographic areas for different population groups.

## Methods

### Study area

This study was undertaken in Anambra state; Southeast Nigeria. The state has a high malaria transmission rate all year round and the annual incidence rate is between 10 to 35%. On the basis of discussions with Anambra State Ministry of Health (MOH) officials, 6 sites were chosen for the study. These were the three largest urban centres (Awka (state capital), Nnewi and Onitsha) from each of the three senatorial zones and one rural LGA randomly selected from each senatorial zone (Njikoka, Aguata and Ogbaru).

Then, one community from each of the three rural LGAs: Enugwu-Ukwu (Njikoka LGA), Ekwulobia (Aguata LGA) and Okpoko (Ogbaru LGA) was selected using two-stage sampling, by first stratifying the communities according to whether they have a general hospital and then randomly selecting the sites from those that have general hospitals

#### Sampling and sample size

The software for population survey in EPI Info 6 was used for sample size calculation. The parameters that were used for sample size calculation were a power of 80%, 95% confidence level and considering 2% as the proportion of people with malaria that used services from the least commonly visited providers (community health workers [20] for first treatment of malaria. The calculations assumed that all the socio-economic groups used the services equally. The last parameter was the study population, which was the number of people with malaria in the study sites. Anambra MOH estimates an average of 6% monthly malaria incidence rate in the state. Hence, using the minimum projected population of each rural site at 30,000 people and each urban area at 60,000 people, it is estimated that a minimum of 1800 and 3600 people will have malaria monthly in each rural and urban area. The calculated minimum sample sizes for the pooled data of urban sites was 720 (240 per urban site) and pooled data for rural site was 663 (221 per rural site) However making allowance for a 5% refusal rate and ensuring an adequate sample size for data analysis, a total sample size of 1200 in the urban areas (400 per urban site and a total of 1050 in rural areas (350 per rural site) was used (an overall sample size of 2,250).

Two-stage sampling was used to select households in each community where the questionnaire was administered. In the first stage, 20 representative streets (urban areas) and 5 hamlets (rural areas) were randomly selected and the number of households in the selected areas enumerated to produce the household list. In the second stage, households were systematically included at regular intervals down the list, the starting point being chosen at random [21]. Information was obtained from respondents from the selected households. The primary respondent was a female caregiver or in her absence a male head of household in whose absence an adult representative was used.

#### Data collection

A pre-tested interviewer-administered questionnaire was used to obtain information from respondents in the randomly selected households. Local educated residents of each community were recruited and trained as field workers to administer the questionnaire. The respondents were asked to rank and also rate their preferences for improved malaria treatment services. Respondents were given a list of different sources of treatment (home, public and private hospitals, public primary healthcare (PHC) centers, pharmacy shops, patent medicine dealers, trained mothers, herbalists and community health workers (CHWs). Colorful option cards that depicted the different providers were also shown to the respondents to help them in visualizing them and aid their ranking and rating.

The contingent ranking and rating of preferences of consumers for different providers was elicited after the different sources of treatment provision were explained to them. The ranking was done before the rating scale. They were first asked to rank the 3 they most preferred then rate each treatment source from 1 to 10. The ranking allowed respondents to state relative preferences amongst the top 3 and in each rating multiple options could be scored at the same level of preference. The questionnaire was also used to collect data on the general socio-economic and demographic characteristics of the respondents and their households, expenditures on food as well as value of home produced and consumed food, and their asset holdings (additional file 1).

### Data analysis

Tabulations, testing of means and non-parametric tests were the major data analytical procedures used. In equity analysis, a SES index was used to categorize the households into SES quartiles: least poor, poor, very poor and poorest. Principal components analysis (PCA) was used to generate the SES index [22,23] that was used to investigate the SES differences in preferences. The input to the PCA was information on ownership of key assets such as a motorcar, a motorcycle, a radio, a refrigerator, a television set, a grinding machine and a bicycle together with the cost of food. In the bivariate analysis the index was analysed as a categorical variable (divided into quartiles), with the ratio of the lowest SES to the highest SES computed as the measure of inequity. Comparison of data between urban and rural area was used to test for geographic differences in preferences. Equity ratios were computed to show the level of difference between the urban and rural areas and between the highest and lowest SES quartile. Chi-square test through cross-tabulations was used to test for relationship of the preferences with SES and geographic location respectively.

Ethical Clearance: The authors received the approval of the ethics committee of the College of Medicine, University of Nigeria, Enugu Campus before carrying out this study.

## Results

### Socio-economic and demographic characteristics of the respondents from the household survey

Table 1 shows that most of the respondents were the wives, followed by male household heads. Hence, most respondents were females, married and middle-aged. The number of household residents ranged from 4.9 in rural to 5.7 in urban, but was 5.3 from the combined data from the six communities. Most of the respondents had some formal education and the average number of years that they spent in school was 10 years. The predominant occupation of the household heads was petty trading.

**Table 1:** Socio-economic and demographic characteristics of the respondents from the household survey

	Urban n = 1260 n(%)	Rural n = 1235 n(%)	Combined n = 2495 n(%)
Status of respondent in household			
• Female household head	96(7.6)	154(12.5)	250(10.0)
• Male household head	211(16.7)	184(14.9)	395(15.8)
• Wife	764(60.6)	708(50.7)	1472(60.0)
• Grandmother	44 (3.5)	44(3.6)	70(2.8)
• Representative	145(11.5)	145(11.7)	306(12.3)
Number of household residents	5.7(2.31)	4.9(2.20)	5.3 (2.3)
Age of respondent: Mean (SD)	40.6(14.06)	41.3(15.4)	41.0(14.8)
Sex (females)	1002 (79.5)	1030 (83.4)	2032 (81.4)
Attended school: n (%)	1130 (89.7)	1009 (81.7)	2139 (85.7)
Years of education: Mean (SD)	10.1 (3.9)	9.9 (3.6)	10.0 (3.7)
Whether married: n %	977 (77.5)	831(67.3)	1735(69.5)
Occupation of household head			
Farmer	42 (3.3)	132(10.7)	174(6.9)
Petty trading	449 (35.7)	412(33.4)	861(34.5)
Govt worker	179 (14.2)	76(6.2)	255(10.2)
Private sector	64 (5.1)	45(3.6)	109(4.3)
Medium/big business	111 (8.8)	76(6.2)	187(7.4)
Self-employed professional	179 (14.2)	117(9.5)	296(11.8)
Unemployed	207 (16.4)	267(21.6)	474(18.9)

### Ranking and Rating of preferences for treatment of malaria

Table 2 shows that public hospitals were ranked highest as preferred source for improved treatment of malaria, which is then followed by training of mothers. The results generally show similar pattern to the rating of preferences with training of patent medicine dealers (PMDs) and use of traditional healers representing the least ranked intervention method.

**Table 2:** Ranking of preferences for treatment of malaria

Providers	Ranking of preferences to best improve treatment n (%)
Public hospitals	761 (30.5%)
Train mothers	474(19.0%)
PHC centres	451(18.1%)
Community health workers	307 (12.3%)
Private hospital	255 (10.2%)
Train PMD and shopkeepers	104 (4.2%)
Traditional healers	121 (4.8%)

Table 3 shows that as in the case of ranking, public hospitals and primary health care centers were rated highest as the preferred means of improving malaria treatment services. Training of mothers was rated 4.3 on the average with a median of 4.0.

**Table 3:** Rating of preferences for Malaria treatment

Providers	Rating of preferences to improve treatment of malaria	
	Mean (SD)	[Median]
Public hospitals	5.35 (1.60)	[6.0]
PHC centres	4.87 (1.61)	[5.0]
Train mothers	4.30 (1.96)	[4.0]
Community health workers	4.17 (1.78)	[4.0]
Private hospital	3.82 (1.94)	[4.0]
Train PMD and shopkeepers	3.20 (1.62)	[3.0]
Traditional healers	2.23 (1.82)	[1.0]

### Geographic and Socio-economic status differences in ranking of preferences for treatment of malaria

Table 4 shows significant (p < 0.05) SES differences in ranking of preferences for some of the malaria treatment provision services. The preference for public hospital was highest for Q4 compared to others, whilst preferences for herbalist decreased as SES class increased. The use of PMDs and training of mothers were ranked higher by poorer quartiles compared to the least poor quartile.

**Table 4:** Socio-economic status (SES) and rural/urban differences in ranking of preferences for treating malaria

	CHW	PHC centre	Public Hospital	Train mothers	Patent medicine dealer	Herbalists	Private hospital
	n (%)	n (%)	n (%)	n (%)	n (%)	n (%)	n (%)
**SES**							
Q1 most poor	66(10.8)	107(17.4)	196(31.9)	123(20.0)	17(2.8)	41(6.7)	62(10.1)
Q2 very poor	78(12.7)	99(16.1)	167(27.2)	141(22.9)	40(6.5)	33(5.4)	57(9.3)
Q3 poor	82(13.3)	120(19.5)	153(24.8)	119(19.3)	31(5.0)	26(4.2)	81(13.2)
Q4 least poor	79(13.0)	123(20.2)	225(37.0)	90 (14.8)	15(2.5)	20(3.3)	55(9.1)
Equity (Q1:Q4) ratio	0.83	0.86	0.86	1.35	1.12	2.03	1.11
Chi-square(p-value	2.3(.52)	4.4 (.22)	25.3(<.01)	13.3(.004)	16.8(.001)	8.4(.038)	7.1(.07)

**R/U**							
Rural	142(11.5)	207(16.8)	399(32.4)	232(18.9)	549(4.4)	77(6.3)	115(9.4)
Urban	165(13.2)	244(19.5)	362(28.9)	242(19.3)	50 (4.0)	44(3.5)	140(11.2)
Equity (R:U) ratio	0.87	0.86	1.12	0.98	1.10	1.80	1.84
Chi-square (p-value	1.6 (.21)	3.0(.086)	3.6 (.059)	.09(.76)	.24(.63)	10.0(.002)	2.3(.13)

## Discussion

Public hospitals were ranked and rated the most preferred choice for the improvement of malaria treatment services in both the higher socio-economic status (SES) group and rural areas. The fact that majority of the respondents stated that the best way to improve malaria treatment services in their community was to improve the quality in services being rendered by public hospitals, might be because public hospitals are known to have a large array of specialist services. Training of mothers that was also highly preferred will help to improve the treatment of malaria. This is line with the Roll Back Malaria (RBM) target for provision of timely and appropriate treatment of malaria [3]. The use of role model mothers has been adopted as one of the strategies for improving the treatment of malaria in Nigeria and in many sub-Saharan African countries.

The SES of the individual influenced their preferences. The finding that the preference for public hospital was highest for the least poor SES (Q4) compared to others may be because the better-off quartiles are usually more educated, and thus have more information about the advantages of having treatment in a public hospital where there is usually a presence of qualified personnel. Herbalists must have been viewed as inferior goods, where preferences fall as SES increases as was alluded to by their low preference as SES increased. The finding that the use of PMDs was ranked higher by poorer quartiles compared to the least poor quartile for improving malaria treatment is most likely due to the fact that it might be cheaper and easier for the poorer SES to visit PMDs. This corroborates the findings of some studies which showed that the poorer households were more likely to seek treatment from low level and informal providers rather than use the hospitals [12,24].

The geographic differences in consumer preferences for providers which showed that herbalist were ranked higher in rural areas than in urban may be because of more familiarity and higher levels of availability of herbalists in the rural areas when compared to the urban areas. The higher preference of public hospitals in the rural area compared to the urban area suggest that those in the rural areas actually need increased availability of public hospitals which would invariably have more qualified health workers and would most likely provide good quality malaria treatment services.

It was seen that the rating scale and ranking of preferences produced similar findings, which is a sign of convergent validity of the findings [25]. Apart from preferences for training of mothers and use of Primary Health care (PHC) centres that switched between 2nd and 3rd most preferred treatment sources, the preferences for the other treatment sources mirrored themselves on both measurement scales, implying that the findings are convergent valid and really represent well thought-out preferences of the consumers. Hence, either can be validly used to determine preferences for malaria treatment.

A limitation of the study was the fact that only two risk factors, namely socio-economic status and geographic locations were explored in this study because the study was primarily concerned with issues of socio-economic and geographic equity. However, other factors such as parity, occupation and age may also affect the preferences of consumers for different providers. The exploration of the role of these other factors should be the focus of future studies. Also, the study did not have a qualitative component, which would have helped to generate qualitative data that will either strengthen or disprove the quantitative findings. Another possible limitation is that use of local residents to act as fieldworkers might bias the results, but regular quality assurance by the investigators ensured that data collected was of very good quality.

## Conclusion

All in all, the paper has shown that preferences for provision of improved malaria treatment services were influenced mostly by SES and also by geographic location of the respondents to a lesser extent. The reasons for the differences in preferences were not explored in the study, but could be as a result of prior knowledge, experiences, costs and availability of the providers. However, it was obvious that people mostly preferred that improved malaria treatment services should be delivered through public health facilities such as hospitals and PHC centers. Hence, there should also be re-invigoration of public facilities for appropriate diagnosis and treatment of malaria, in addition to improving the financial and geographic accessibility of such facilities. However, there is a role of home management of malaria through training of mothers. However, home management will not work if the quality of services rendered by providers where drugs for home management are purchased are not improved. Therefore, there is the need for the government and development partners to also improve the quality of services of PMDs and pharmacy shops so that there is a holistic improvement of malaria treatment services.

## Competing interests

The authors declare that they have no competing interests.

## Authors' contributions

OO conceived the study. All the authors participated in data collection and analysis. NU wrote the manuscript with input from all the authors

## Pre-publication history

The pre-publication history for this paper can be accessed here:

http://www.biomedcentral.com/1471-2458/10/7/prepub

## Supplementary Material

Additional file 1 **Contains a household questionnaire on the preferences of different household for where they sought treatment for malaria treatment.** It also contains the socio-demographic detail of each respondent and their socio-economic status based on household owned assets and food expenditure pattern. Click here for file
