# Supplementary material for: Do consumers' preferences for improved provision of malaria treatment services differ by their socio-economic status and geographic location? A study in southeast Nigeria
Source: BMC Public Health. 2010 Jan 5;10:7. doi: 10.1186/1471-2458-10-7 (PMC3397432; doi:10.1186/1471-2458-10-7)
Supplement: Additional file 1 — Contains a household questionnaire on the preferences of different household for where they sought treatment for malaria treatment. It also contains the socio-demographic detail of each respondent and their socio-economic status based on household owned assets and food expenditure pattern. [file 1471-2458-10-7-S1.DOC]

## Abridged Consumer Questionnairt

## Dear respondent,

Please sign this consent form to acknowledge participation in the study. ***(Interviewer: See last page for consent form. Give it to the respondent to sign).***

Please, enter the appropriate number representing the answer given in the spaces provided

***Section 2: Preferences and Contingent Ranking for Different Providers for the Improved Treatment of Malaria***

In Section 2, before we ask you about your specific experience with malaria, we will first like to determine the best treatment strategy that you prefer for the treatment of malaria in your community.

**THE OPTIONS**

**Introducing community-based health workers (or village health workers):** Some knowledgeable community members will be trained to become community-based health workers and they will treat malaria with appropriate doses of genuine drugs. People will be required to pay for treatment you receive from the health workers.

**Improving the quality of malaria treatment services in government-owned primary health care centers:** The quality of laboratory diagnosis and availability of genuine drugs for treating malaria in health centers will be improved through government health policy intervention.

**Improving the quality of malaria treatment services in government-owned general hospitals:** The method for laboratory diagnosis and the ready availability of genuine drugs for treating malaria in the facilities will be improved.

**Educating mothers on how to self-treat:** The mothers living within your community will be taught by trained health workers on how to recognize and properly treat malaria in their households with genuine drugs. The mothers will however have to buy the drugs from patent medicine dealers or pharmacy shops.

**Train shopkeepers and patent medicine dealers on proper drug prescription (chemists):** The chemists will be taught by trained health workers about how to recognize and properly treat malaria with genuine drugs.

**Improving treatment in private clinics and hospitals and laboratories:** The private hospitals, clinics and laboratories will continue treating as they currently do.

15. How best do you think treatment of malaria can be improved in your community?

1=yes 0=No *[Enumerator: Read out all the options and the respondent should select threemost preferred options. In case the respondent has difficulty understanding any of the options, feel free to read out the options from above.]*

15a. Introducing community-based health workers (or village health workers) [ ]

15b. Improving the quality of services in government healthcare facilities [ ]

15c. Educating mothers on how to self-treat [ ]

15d. Improving the quality of care in health centres [ ]

15e. Train shopkeepers and patent medicine dealers on proper drug prescription (chemists) [ ]

15f. Train herbalists to use tablets to treat malaria [ ]

15g. Improving services in private healthcare facilities [ ]

15h. Others [ ] (specify) ______________________________________

**REMINDER! *[Instruction: enumerator, ask the respondent whether he or she has any question and to recollect all the different treatment options]***

16. If you had all of the above options available to you, please rate them according to your preference. I will now read and explain the available six options to you. *[Enumerator: Read out all the options to the respondent]*

Please, rate how you prefer the different treatment choices. Rating is from 1 (least preferred) to 10 (most preferred). SO YOU CAN RANK 1,2,3,4,5,6,7,8,9,10

16a. Introducing community-based health workers (or village health workers) [ ]

16b. Improving the quality of malaria treatment services in government-owned primary health care centers [ ]

16c. Improving the quality of malaria treatment services in government-owned general hospitals [ ]

16d. Educating mothers on how to self-treat [ ]

16e. Train shopkeepers and patent medicine dealers on proper drug prescription (chemists) [ ]

16f. Improving treatment in private clinics and hospitals and laboratories [ ]

***Section 7: Weekly food costs and asset holdings***

This section is designed to find out information to determine your socio-economic status

109. How much did your household spend to purchase food from the market in the past one week on the various items that I will read out?

| Item | Quantity | Who purchased | Amount |
| --- | --- | --- | --- |
| Gari  Beans  Cassava (akpu)  Rice  Corn  Fish  Meat  Vegetables  Others (specify)  Total |  |  |  |

110. If the food items that your household produced, but also consumed in the past one week were bought from the market, how much will they cost?

| Item | Quantity | Amount | **Total** |
| --- | --- | --- | --- |
| Gari  Beans  Cassava (akpu)  Rice  Corn  Fish  Meat  Vegetables  Others (specify)  Total |  |  |  |

111. Total food cost (*Enumerator add 109+ 110)* [ ] Naira

112. Does anyone in the household own any of the following? 1 = yes 0 = no

112a. Radio [ ]

112b. Television [ ]

112c. Air conditioner [ ]

112d. Bicycle [ ]

112e. Motorcycle [ ]

112f. Car [ ]

112g. Fridge [ ]

THANK YOU

# 
